# Incidence, microbiological aspects and associated risk factors of catheter-related bloodstream infections in adults on chronic haemodialysis at a tertiary hospital in Uganda

**DOI:** 10.1016/j.ijregi.2022.09.002

**Published:** 2022-09-14

**Authors:** Doreen Nanyunja, Mogamat-Yazied Chothia, Kenneth C. Opio, Ponsiano Ocama, Freddie Bwanga, Daniel Kiggundu, Pauline Byakika-Kibwika

**Affiliations:** a Department of Medicine, Makerere University College of Health Sciences, Kampala, Uganda; b Division of Nephrology, Department of Medicine, Faculty of Medicine and Health Sciences, Stellenbosch University, Cape Town, South Africa; c Aga Khan University Hospital, Nairobi, Kenya; d Department of Immunology and Molecular Biology, Makerere University College of Health Sciences, Kampala, Uganda

**Keywords:** Haemodialysis, Central venous catheter, Bloodstream infection, Nosocomial, Antibiotic, Sub-Saharan Africa

## Abstract

**Objectives:**

The high burden of infectious complications among patients receiving haemodialysis (HD) via central venous catheters increases morbidity and mortality. This study determined the incidence of catheter-related bloodstream infections (CRBSIs), microbiological profile of causative organisms, and associated predictors in patients on chronic HD.

**Methods:**

A prospective single-centre cohort study of 121 adult patients with end-stage kidney disease was conducted from October 2019 to March 2020. Antibiotic susceptibility was determined by the Kirby–Bauer disk diffusion method. Cox proportional hazards model was used to determine predictors of CRBSI.

**Results:**

The mean age was 50 (standard deviation 14.9) years and the median duration of follow-up was 69 (interquartile range 23–124) days. At least one CRBSI was recorded for 41% of patients, at a rate of 5.2 infections per 1000 patient-days. Causative organisms were predominantly Gram-negative bacteria (60.3%), and 36.5% of all isolates were multi-drug resistant. Anaemia [hazard ratio (HR) 5.44, *P=*0.019, 95% confidence interval (CI) 1.32–22.48] and previous bloodstream infection [HR 2.47, *P=*0.028, 95% CI 1.10–5.54] were predictors of CRBSI.

**Conclusion:**

The high incidence of CRBSI in patients on chronic HD with predominance of Gram-negative bacteria means that catheter care bundles should include Gram-negative coverage.

## Highlights

• Infections increase mortality and morbidity in patients with kidney disease.
• Central venous catheters for haemodialysis increase infectious complications.
• Gram-negative isolates dominate catheter-related bloodstream infections (CRBSIs).
• Prior bloodstream infections and anaemia are significant risk factors for CRBSIs.

## Introduction

Chronic kidney disease (CKD) affects 10% of the world's population, with projections showing greater increments in resource-limited settings such as sub-Saharan Africa compared with high-income countries (Hill et al., 2016; Xie et al., 2018). Recent community-based studies in East Africa reported the prevalence of CKD to be as high as 21% (Kalyesubula et al., 2017; Muiru et al., 2020). As a result, the frequency of end-stage kidney disease (ESKD) and subsequent demand for life-saving kidney replacement therapy (KRT) is rising in Africa (Arogundade et al., 2020). However, only 10% of adults living in sub-Saharan Africa have access to KRT. Many patients die within 6 months of the initiation of haemodialysis (HD), which may be related to, among others, vascular-access-related complications (Halle et al., 2016; Ashuntantang et al., 2017).

Patients receiving HD through central venous catheters (CVCs) are at high risk of bloodstream infections (BSIs) compared with patients receiving HD via arteriovenous fistulae or grafts (Taylor et al., 2002; George et al., 2006). Infections are a major contributor to hospitalization and death among HD patients, accounting for 20% and 36%, respectively (Akoh, 2011; Dalrymple et al., 2012; Collins et al., 2015). CVCs used in HD are a major risk factor for BSI, with incidence rates of CRBSI ranging from 3.25 to 10.8 episodes per 1000 patient-days for temporary CVCs and from 0.55 to 4.4 episodes per 1000 patient-days for permanent CVCs (Fysaraki et al., 2013; Patel et al., 2013; Sahli et al., 2017; Brown et al., 2018; Shalaby et al., 2018; Agrawal et al., 2019; Zanoni et al., 2021). Additional risk factors for BSI in HD patients include comorbidities such as diabetes, uraemia, iron overload, older age and anaemia (Taylor et al., 2002; James et al., 2008).

Gram-positive bacteria such as *Staphylococcus aureus* and coagulase-negative staphylococci are the most commonly implicated organisms responsible for CRBSIs in HD patients, and collectively account for up to 80% of isolates (Lok and Mokrzycki, 2011). Gram-negative bacteria have been identified less frequently, especially during outbreaks (Agrawal et al., 2019; Novosad et al., 2019). Of concern is the rising rate of multi-drug-resistant (MDR) pathogens, often related to the inadequate and inappropriate use of antimicrobial drugs, and associated with increased healthcare costs and poorer patient outcomes (Diekema et al. 2019; Vanegas Múnera and Jiménez Quiceno 2019).

Due to the paucity of data from Africa, this prospective cohort study was conducted in order to investigate the incidence of CRBSIs, identify responsible micro-organisms, and determine their antimicrobial resistance profiles and associated predictors of CRBSI among patients who received chronic HD via CVCs.

## Methods

### Study design and setting

This prospective cohort study was conducted at the HD unit of Kiruddu National Referral Hospital, a public tertiary hospital in Kampala, Uganda, from 1 October 2019 to 31 March 2020. The study cohort included adults with ESKD who were receiving chronic HD via CVCs during this period, and were observed from the time of enrolment to confirmed CRBSI or study end date.

### Description of local practice

The HD unit of Kiruddu Hospital has 20 beds with an average daily occupancy of 40 patients (both in- and outpatients), most of whom have twice-weekly dialysis sessions, each lasting for 4 h hours. Silicone double-lumen tunnelled cuffed CVCs (Permacath) were inserted by either a nephrologist, a general practitioner or a trained dialysis nurse. The insertion procedure followed the aseptic technique including hand hygiene, appropriate sterile barrier measures, and skin preparation with 70% alcohol solution followed by povidone iodine. The right internal jugular vein was the preferred site, with femoral access reserved for those in whom internal jugular venous catheterization was not possible. Insertion was done blindly under local anaesthesia. Prior to connection to the dialysis machine, catheter hubs and exit sites were disinfected with 70% alcohol. After each HD session, CVCs were filled with 2 mL of unfractionated heparin sodium (1000 IU/mL), and thereafter the catheter ports were disinfected with 70% alcohol solution, and dressed with sterile gauze covering and semipermeable adhesive dressing. Used blood lines and dialysers were removed and disposed of in infectious waste bins after HD. A hot water rinse with sodium hypochlorite was used to disinfect the machines between patients, and a citrate rinse was done at the end of each day.

Trained nurses assessed the patients for clinical features of infection during each HD session, and those with a high index of suspicion had blood sampling performed using the guidelines of the Centers for Disease Control and Prevention for collecting blood culture specimens (CDC, 2018). The blood culture set comprised two (8–10 mL) samples drawn simultaneously: one from the CVC and the other from a separate venepuncture site on another limb or the extracorporeal HD circuit, in accordance with current international guidelines (Mermel et al., 2009).

All patient microbiological samples were sent to a single laboratory located outside of the hospital. The unit had no vascular access infection protocol, so the choice of empirical antibiotics and the decision to remove a CVC following confirmation of CRBSI was left at the discretion of the attending nephrologist. However, due to cost limitations, catheter salvage was attempted in most cases of CRBSI.

### Participants and data sources

Using administrative records, patients aged ≥18 years with ESKD on HD who were willing to provide informed consent to participate in the study were identified. Patients who received HD via arteriovenous fistula, as well as transient patients who were expected to be managed at the facility for <4 weeks due to temporary travel or transfer from another HD unit within Uganda or another country, were excluded. Baseline information on patient demographics (age, sex and employment status), length of time on dialysis and number of HD sessions per week, comorbidities (diabetes mellitus, human immunodeficiency virus and cancer), history of BSI since HD initiation, and current medications [iron supplementation and intravenous (IV) antibiotics] was obtained through direct enquiry and use of the patients’ medical records.

### Exposure and case definitions

Exposure was defined as receiving HD via CVC from the study unit from the time of enrolment into the study. As most patients had a CVC in place at the beginning of the study period, their start date for computing patient days at risk began on 1 October 2019, the first day of the study. The suspicion of bacterial infection among patients was based on clinical definitions from the National Healthcare Safety Network dialysis event manual (NHSN, 2013) as the presence of one or more of: axillary temperature <36° C or >37.8 °C or history of fever and/or chills, symptoms and signs of infection such as purulent discharge from CVC exit site, malaise, alteration in mental status or unexplained hypotension (systolic blood pressure <90 mmHg).

CRBSI was defined as positive blood culture with concurrent exit site or tunnel infection, or without another identifiable source of infection (Mermel et al., 2009).

### Organism identification

Blood culture samples were inoculated into BACTEC aerobic plus resin media bottles and subsequently placed in a BACTEC 9120 machine (BD Diagnostics, Franklin Lakes, NJ, USA). Positive growths were Gram-stained and subcultured on to blood agar and MacConkey agar as indicated for species identification and typing. Subsequent drug sensitivity testing was done on Mueller–Hinton agar using the Kirby–Bauer disk diffusion method, while antimicrobial panels were selected for testing based on the guidelines of the Clinical and Laboratory Standards Institute (CLSI, 2018).

Bacteria causing BSIs were classified as Gram-negative, Gram-positive or common contaminants. Bacteraemia was considered polymicrobial if more than one species of micro-organism was isolated in the same blood culture. Susceptibility to antibiotics was reported as susceptible, intermediate, resistant or not available. MDR bacteria were defined as bacteria that had acquired non-susceptibility to at least one agent in three or more antimicrobial categories (Magiorakos et al., 2012).

### Statistical analysis

All analyses were performed using STATA Version 16 (StataCorp LLC, College Station, TX, USA). For calculation of the CRBSI incidence rate, the first event for each patient during the study period was considered as the numerator, and the total cumulative number of days at risk for the same period (patient-days) was considered as the denominator. These rates were expressed as number of infections per 1000 patient-days. Microbiological analyses were performed using Clinical and Laboratory Standards Institute antimicrobial susceptibility testing and reporting guidelines (CLSI, 2018).

Baseline characteristics were displayed as count and percentage for categorical variables, and as mean and standard deviation (SD) for continuous variables if data were normally distributed. Where continuous variables were not normally distributed, median and interquartile range (IQR) were used. Student's *t*-test was used to compare continuous variables that were normally distributed, while Mann-Whitney *U*-test was used for continuous variables that were not normally distributed. Chi-squared test and Fisher's exact test was used to assess differences between categorical variables in patients who had CRBSIs and those who did not. Cox multi-variate proportional hazards model was used to determine the association between predictor variables, including age (>60 years), length of time on dialysis, previous BSI and anaemia, with CRBSI as the dependent variable. *P*<0.05 was considered to indicate significance, and 95% confidence intervals (CI) were used.

### Ethical considerations

Ethical approval was obtained from Makerere School of Medicine Research and Ethics Committee (REC REF 2019/100), and administrative clearance was obtained from Kiruddu National Referral Hospital. Written informed consent was obtained from patients prior to study enrolment.

## Results

**Figure 1:**
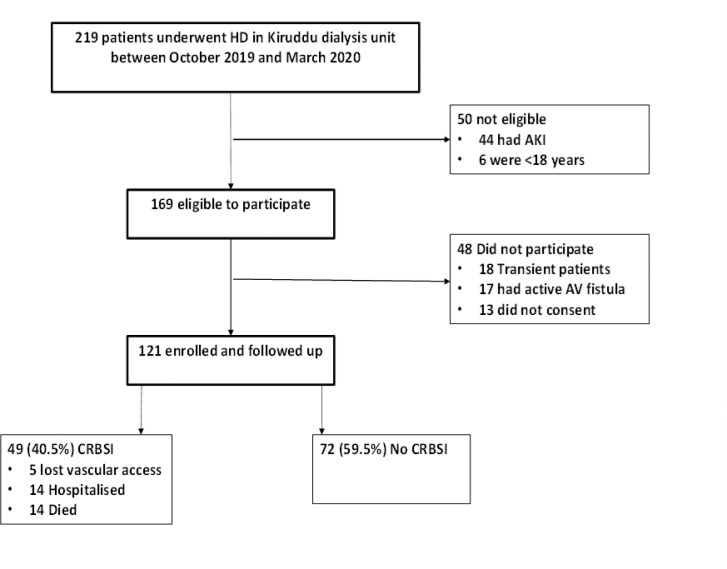
Patient flow diagram. HD, haemodialysis; AKI, acute kidney injury; AV, arteriovenous; CRBSI, catheter-related bloodstream infection.

In total, 219 adult patients underwent HD at the study unit over the 6-month period. Of these, 98 patients were excluded because of acute kidney injury (*n*=50), transience (*n*=18), arteriovenous fistula vascular access (*n*=17), or they declined to participate (*n*=13). Therefore, 121 patients were included in the analysis, with a cumulative 9441 patient-days at risk (Figure 1).

The mean age was 50 (SD 14.9) years, and most patients were male [*n*=76 (62.8%)]. More than one-third (42.2%) of patients were unemployed. The median duration of follow-up was 69 (IQR 23–124) days. The mean haemoglobin level was 8.9 (SD 1.99) g/dL, and 118 (97.5%) patients were on supplementary iron (oral, IV or both). Diabetes mellitus was the most common comorbidity [*n*=36 (29.8%)]. Other baseline characteristics are shown in Table 1.

**Table 1:** Baseline sociodemographic and clinical characteristics of patients

Variable	All 121 (100)	With CRBSIs *n*=49 (100)	Without CRBSIs *n*=72 (100)	*P*-value
Age, years, mean (SD)	50 (14.9)	46 (13.8)	52 (15.3)	0.032
Age group, years				
18–59		40 (81.6)	48 (66.7)	0.070
≥60		9 (18.4)	24 (33.3)	
Male sex		31 (81.6)	45 (37.2)	0.932
Length of dialysis, months, mean (SD)	6.7 (11.2)	8.7 (13.3)	5.3 (9.3)	0.099
0–6		30 (61.2)	54 (75.0)	0.106
>6		19 (38.8)	18 (25.0)	
Comorbidity^a^				
Diabetes		12 (24.5)	24 (33.3)	0.296
Cancer		2 (4.1)	1 (1.4)	0.350
Human immunodeficiency virus		2 (4.1)	3 (4.2)	0.982
Clinical and laboratory parameters				
Anaemia (Hb <11.5 g/dL)		47 (95.9)	59 (81.9)	0.022
Previous BSI		7 (14.3)	2 (2.8)	0.018
Supplementary iron^b^		48 (98)	70 (97.2)	0.798

Hb, haemoglobin; BSI, bloodstream infection; SD, standard deviation.

a Some patients had more than one comorbidity.

b Patients received oral or intravenous iron or both.

The most common symptoms reported by patients with suspected infection were chills (85%) and fever (79%), while 70% of patients reported both. Sixty-seven (48.6%) patients were initiated on empirical IV antibiotics for suspected CRBSIs. The most frequently prescribed empiric antibiotics were vancomycin (44.7%) and ceftriaxone (34%). At least one CRBSI occurred in 49 patients, giving an incidence rate of 5.2 BSIs per 1000 patient-days. Five patients had their dialysis catheters replaced because of CRBSIs.

In total, 68 blood cultures were performed; of these, 54 cultures grew recognized pathogens with a positivity rate of 79.4%. Polymicrobial growth occurred in 12/54 (22.2%) of the positive cultures. Overall, more Gram-negative bacteria than Gram-positive bacteria were identified on blood culture (60.3% vs 39.7%, respectively). Details of the isolated micro-organisms are summarized in Table 2.

**Table 2:** Micro-organism species isolated from positive blood cultures

Micro-organism	Number (n=68)	Frequency (%)
Gram-negative bacteria	41	60.3
Acinetobacter spp.	14	20.6
Klebsiella pneumoniae	10	14.7
Pseudomonas aeruginosa	8	11.8
Escherichia coli	7	10.3
Enterobacter spp.	1	1.5
Citrobacter freudii	1	1.5
Gram-positive bacteria	27	39.7
Methicillin-susceptible Staphylococcus aureus	10	14.7
Methicillin-resistant Staphylococcus aureus	10	14.7
Enterococcus spp.	6	8.8
Coagulase-negative staphylococci	1	1.5

**Figure 2:**
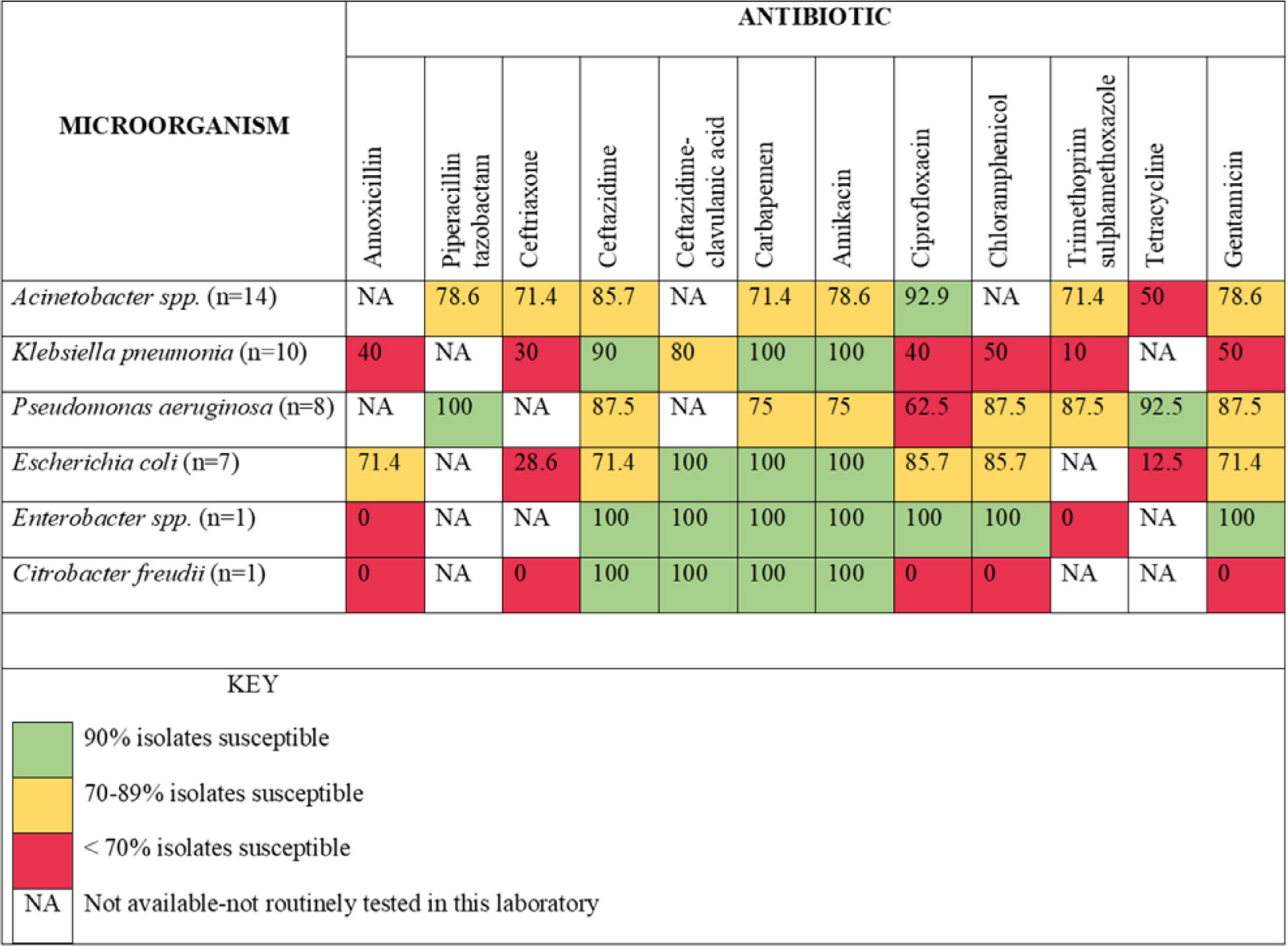
Antibiogram for Gram-negative bacteria.

**Figure 3:**
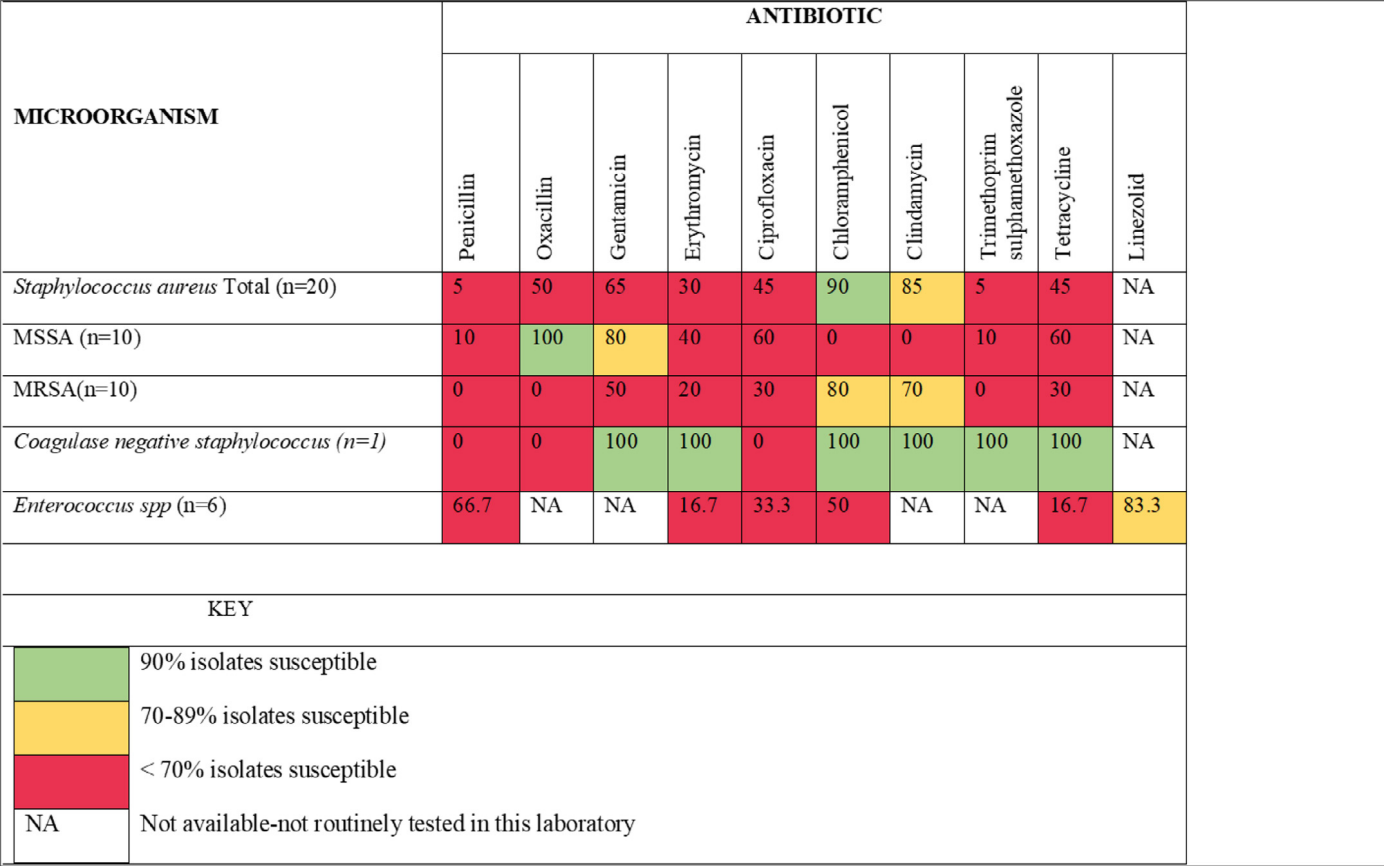
Antibiogram for Gram-positive bacteria.

Twenty-seven (36.5%) micro-organisms were MDR, and the antibiograms for the Gram-negative and Gram-positive bacteria isolated are summarised in Figures 2 and 3, respectively.

Factors associated with CRBSIs on multi-variate analysis included anaemia [hazard ratio (HR) 5.44, 95% CI 1.32–22.48] and a history of BSI [HR 2.47, 95% CI 1.10–5.54] (Table 3).

**Table 3:** Cox regression analysis for risk factors of catheter-related bloodstream infections

Variable	Bivariate analysis		Multi-variate analysis	
	HR (95% CI)	*P*-value	HR (95% CI)	*P*-value
Age group ≥60 years	0.54 (0.25–1.15)	0.110	0.46 (0.22–0.96)	0.038
Sex (male)	1.34 (0.73–2.47)	0.341	–	-
Diabetes (yes)	0.98 (0.57–1.90)	0.951	–	-
Previous BSI (yes)	2.84 (1.12–7.18)	0.027	2.47 (1.10–5.54)	0.028
Anaemia (Hb <11.5 g/dL)	5.86 (1.41–24.36)	0.015	5.44 (1.32–22.48)	0.019
Supplementary iron^a^ (yes)	1.64 (0.22–12.58)	0.629	–	-

Hb, haemoglobin; BSI, bloodstream infection; HR, hazard ratio; CI, confidence interval.

a Patients received oral or intravenous iron or both.

## Discussion

A high incidence of CRBSIs (5.2 episodes per 1000 patient-days) was found in the HD unit. This rate was higher compared with studies than reported rates ranging from 0.55 to 4.4 episodes per 1000 patient-days in patients with permanent CVCs (Fysaraki et al., 2013; Patel et al., 2013; Brown et al., 2018; Zanoni et al., 2021). However, the CRBSI rate in the present study was lower relative to other studies from similar settings. This difference was likely attributable to greater utilization of temporary dialysis CVCs, whereas the population of the present study predominantly used tunnelled cuffed CVCs for chronic HD (Sahli et al., 2017; Agrawal et al., 2019). Temporary dialysis CVCs are known to carry a significantly higher risk of infection compared with tunnelled cuffed catheters in patients on chronic HD (George et al., 2006; Grothe et al., 2010).

Regarding the causative organisms, most (60.3%) CRBSIs were caused by Gram-negative bacteria. While patient characteristics, dialysis infrastructure and existing IPC policies may have contributed to variability in the micro-organisms isolated, the study findings were aligned with global trends that have reported a growing proportion of Gram-negative bacteraemia in patients with indwelling CVCs (Marcos et al., 2011; Braun et al., 2014; Murray et al., 2015). This epidemiological shift may have resulted from a number of factors, including the promotion of CVC care approaches that focus on control of Gram-positive bacterial infections or contamination (Mermel et al., 2009; Schweiger et al., 2015; Miller et al., 2016; Fisher et al., 2020).

The most common Gram-negative organisms were *Acinetobacter* spp., *Klebsiella pneumoniae* and *Pseudomonas aeruginosa*. It is speculated that these infections were largely nosocomial in origin, as has been reported elsewhere (Marcos et al., 2011; Murray et al., 2015; Diekema et al., 2019; Surapat et al., 2020). *Acinetobacter* spp., in particular, were implicated in nosocomial BSIs, especially in association with CVCs (Wisplinghoff et al., 2012). Also, since the HD unit is located within a busy tertiary hospital, contact with hospitalized patients and contaminated hospital surfaces outside of the dialysis unit may have played a role. Additionally, contamination of the hospital water supply and reverse osmosis membranes may be a contributing factor, as recent reports have shown several outbreaks of water-borne Gram-negative bacterial infections in HD units (Dijkshoorn et al., 2007; Maragakis and Perl, 2008; Novosad et al., 2019; Kanamori et al., 2022). Unfortunately, the study unit did not perform routine water quality testing while this study was being conducted, and therefore the possibility of a contaminated water supply is purely speculative.

The significant proportion of CRBSIs attributed to *S. aureus* (29.4%) was not surprising. This has also been reported from other centres in South Africa, Algeria and India (Bisiwe et al., 2015; Sahli et al., 2017; Agrawal et al., 2019). The high prevalence of methicillin-resistant *S. aureus* (MRSA) (50% of *S. aureus* isolates) is comparable with other centres in the region, and reflects the rising trend of MRSA in hospital settings with predominance among patients on KRT (Wangai et al., 2019). These findings emphasize the importance of including vancomycin as part of empirical antibiotic coverage for CRBSIs in the HD unit.

Of particular concern, more than one-third of bacterial isolates were MDR, which may indicate that the infections were of nosocomial origin (Wisplinghoff et al., 2012; Vanegas Múnera and Jiménez Quiceno, 2019). These high MDR rates could be attributed to unregulated prescription of antimicrobials, as previous studies from Uganda have demonstrated that erratic use of antimicrobials was associated with the emergence of MDR bacterial infections (Kiguba et al., 2016; Agaba et al., 2017). These findings highlight the need for development of local BSI treatment protocols within the HD unit, as well as antibiotic stewardship within the hospital to improve the profile of bacterial resistance (Leone and Suter, 2010; Surapat et al., 2020).

Anaemia at baseline was associated with a five-fold higher risk of CRBSI during follow-up, although this estimate was not precise, as indicated by the wide CI, possibly due to the small sample size for this analysis. Anaemia has previously been reported as a risk factor for bacterial infections, and portends poor outcomes in HD patients in both low- and high-income settings (Roberts et al., 2004; Ruggajo et al., 2019). As many patients did not receive the minimum frequency of dialysis per week, it is hypothesized that anaemia in the study population was an indicator of chronic inflammation from poor control of the uraemic milieu, predisposing to immune dysregulation and resultant elevated risk of infection (Leone and Suter, 2010). Additionally, anaemic patients on HD often receive IV iron supplementation, stimulating bacterial growth (Ishida and Johansen, 2014).

Patients who had a prior history of BSI during HD were found to be two-fold more likely to develop a subsequent infection. This was also reported in a large multi-centre study (Hoen et al., 1998). In the present setting, it is postulated that this association may have resulted from the common practice of attempts at catheter salvage, as many patients cannot afford the costs of new CVCs. Biofilms in CVCs have been found to form resistant barriers to antibiotic clearance of pathogenic bacteria (Vanegas Múnera and Jiménez Quiceno, 2019).

### Strengths and limitations

This was a large prospective study; therefore, it is unlikely that patients were missed, and the completeness of the data improved the robustness of the results. However, although this was a large study, the results may not be generalizable due to the participation of a single centre at a national referral hospital. Secondly, the severity of illness was not measured in the patient cohort, and this is acknowledged as an unmeasured confounding factor. Additionally, several patients were already receiving both oral and IV antibiotics prior to blood culture sampling, so the number of CRBSIs may be an underestimate. Finally, the lack of data on the quality of the water system used in the dialysis unit meant that the inferences were purely speculative.

## Conclusion

This study found a high burden of CRBSIs in patients with ESKD receiving chronic HD. These infections may be reduced by implementing a ‘fistula first’ policy. Although empirical use of IV vancomycin is justified in this patient cohort, most infections were due to Gram-negative bacteraemia; as such, initial empirical therapy should include Gram-negative bacterial cover. Future research should focus on the source of the Gram-negative bacteraemia and tailored catheter care bundles, so that interventions can be implemented to reduce the rate of these life-threatening infections.

## Conflict of interest statement

None declared.
